# First Record of *Dioryctria simplicella* (Lepidoptera: Pyralidae) in China: Morphology, Molecular Identification, and Phylogenetic Position

**DOI:** 10.3390/insects16070664

**Published:** 2025-06-26

**Authors:** Niya Jia, Xiyao Ding, Dan Xie, Huanwen Chen, Defu Chi, Jia Yu

**Affiliations:** The Key Laboratory of Sustainable Forest Ecosystem Management-Ministry of Education, College of Forestry, Northeast Forestry University, Harbin 150040, China; jianiya9937@126.com (N.J.); dxyemail2025@126.com (X.D.); xiedan0807@126.com (D.X.); chenhuanwen2020@126.com (H.C.)

**Keywords:** *Dioryctria simplicella*, *Pinus sylvestris* var. *mongolica* Litv., new record, Lepidoptera, Pyralidae, cone pest

## Abstract

*Dioryctria* Zeller, 1846 (Lepidoptera: Pyralidae) is a significant genus whose species primarily infest coniferous trees and are predominantly distributed across the Northern Hemisphere. To date, 17 species within this genus have been recorded in China. This study reports the discovery of *Dioryctria simplicella* (Heinemann, 1863) in China. During field surveys in forests of Heilongjiang Province, *D. simplicella* was observed infesting the cones and trunks of *Pinus sylvestris* var. *mongolica* Litv. as larvae. Comprehensive morphological descriptions and diagnostic characteristics of the adult, larva, pupa, and egg stages of *D. simplicella* are provided herein to facilitate accurate species identification within the genus. Molecular phylogenetic analysis based on mitochondrial cytochrome c oxidase subunit I (COI) DNA barcoding sequences was conducted to assess the phylogenetic position of *D. simplicella* within *Dioryctria*. These results strongly support its species identity and clarify its phylogenetic relationships with congeners. This discovery not only expands the known diversity of Lepidoptera in China but also provides new data supporting taxonomic and phylogenetic studies of the genus *Dioryctria*.

## Simple Summary

The study reports on the occurrence of *Diocrytria simplicella* Heinemann, 1865, a potential forestry pest, in China for the first time. Based on detailed morphological comparisons, we provide a systematic description of this species, including key diagnostic characters of the adult external features and genitalia structures. Additionally, molecular identification based on the mitochondrial COI gene, supported by phylogenetic analysis, further corroborates its taxonomic placement within the genus *Dioryctria*. This work expands the known distribution of *D. simplicella* and contributes to the understanding of its systematics.

## 1. Introduction

The genus *Dioryctria* (Lepidoptera: Pyralidae), established by Zeller in 1846, is primarily distributed across the Holarctic region, with a few species extending into the northern tropics [1,2]. The larvae of the *Dioryctria* genus feed on various coniferous trees and are significant forest pests [3]. They typically bore into the trunks, new shoots, and cones of *Pinus*, *Picea*, *Abies,* and *Larix* species, causing substantial damage to tree growth, timber quality, seed production, and landscape value [4,5,6,7,8,9]. To date, 86 *Dioryctria* species have been documented worldwide, with 12 species groups currently recognized based on morphological characteristics [4,10,11,12,13,14,15,16]. There are 17 *Dioryctria* species recorded in China [13,17,18,19,20,21], including *D. abietella* [Denis & Schiffermüller, 1775], *D. castanea* [Bradley, 1969], *D. fanjingshana* [Li, 2009], *D. magnifica* [Munroe, 1958], *D. auloi* [Barbey, 1930], *D. kunmingnella* [Wang & Sung, 1985], *D. mendacella* [Staudinger, 1859], *D. mongolicella* [Wang & Sung, 1982], *D. pryeri* [Ragonot, 1893), *D. reniculelloides* [Mutuura & Munroe, 1973], *D. resiniphila* [Segerer & Pröse, 1997], *D. rubella* [Hampson, 1901], *D. schutzeella* [Fuchs, 1899], *D. splendidella* [Herrich-Schäffer, 1848), *D. sylvestrella* [Ratzeburg, 1840], *D. yiai* [Mutuura & Munroe, 1972], and *D. yuennanella* [Caradja, 1937].

The taxonomic status of *Dioryctria simplicella* Heinemann, 1865 has been debated for over a century, with the loss of its type specimens exacerbating doubts regarding its species validity and identity. In 1901, Rebel [22] removed *D. simplicella* from genus *Dioryctria* and placed the species in synonymy of *Salebriopsis albicilla* (Herrich-Schäffer, 1849). In 1968, Roesler [23] reinstated its status as a valid species and name within the genus *Dioryctria*. In 1980, Petersen and Gaedike [24] regarded both *D. simplicella* and *D. mutatella* (Fuchs, 1899) [25] as synonymous, but they expressed reservations about the species identity Heinemann originally described. The advent of DNA barcoding using the cytochrome c oxidase subunit I (COI) gene [26,27,28] provided new tools for resolving this taxonomic uncertainty. Through integrated morphological and mitochondrial COI sequence analysis, Knölke [16] conclusively demonstrated that *D. simplicella* as it is universally understood represents a melanistic form of *D. mutatella*, thereby confirming their synonymy.

This study reports the first country record of *Dioryctria simplicella* in China. Its taxonomic status was confirmed through integrated evidence from classical morphological identification and mitochondrial COI gene sequence analysis. We carefully compared diagnostic characteristics of adult external morphology and genitalia, larvae, pupae, and eggs. Molecular identification involved sequencing the COI gene of *D. simplicella*. Based on genetic distance thresholds and phylogenetic tree topology, its status as a distinct species was validated. *D. simplicella* was historically subject to taxonomic controversy with *D. mutatella* due to the loss of type specimens and melanistic phenotypic variation. This study identified stable diagnostic traits in Chinese populations, supporting it as a separate species, and its discovery provides a critical addition to the East Asian fauna.

## 2. Materials and Methods

### 2.1. Specimens Collection

The specimens were collected from a 20-year-old *Pinus sylvestris* var. *mongolica* Litv. forest at the Xinjiang Experimental Forest Farm (124.46° N, 48.04° E), Qiqihar City, Heilongjiang Province, China, between 20 May and 30 September 2024. Adult and larval stages collected in the field were transported to the laboratory, where eggs and pupae were obtained. Adult were pinned and stored in dry condition. Specimens (larvae, pupae, eggs) were preserved in 95% ethanol at −20 °C. Morphological examination and DNA barcoding were employed for identification. Voucher specimens are deposited in the Northeast Forestry University (NEFU) Entomological Specimen Room (Harbin, China) and are accessible upon request. Related laboratory work was conducted at NEFU.

### 2.2. Morphological Study

Following immersion in 10% NaOH solution for 12–24 h, adult abdomens were tissue cleared and genitalia were dissected. Stereomicroscopic imaging of genitalia and eggs employed a Canon A620 camera (Canon, Tokyo, Japan), whereas macrophotography of whole specimens utilized a Canon EOS R5 camera (Canon, Tokyo, Japan). All photographs and images were processed in Adobe Photoshop CS6 v.13.0. Morphological descriptions of adults followed the methodology of Kuang and Li [19], while morphological descriptions of larvae followed the method of Piao et al. [29].

### 2.3. Molecular and Phylogenetic Study

Molecular analysis was performed using the standard mtDNA COI barcoding region on two larvae and four adults (two males and two females). DNA extraction from adult legs and intact larvae was performed with the TIANamp Micro DNA Kit (TIANGEN, Beijing, China) following standardized procedures. PCR amplification of the mitochondrial cytochrome c oxidase subunit I (COI) gene utilized universal primers as follows: LCO1490: 5′-GGTCAACAAATCATAAAGATATTGG-3′, HCO2198: 5′-TAAACTTCAGGGTGACCAAAAAATCA-3′ [30]. The PCR reaction was performed in a total 25 µL volume: 0.5 µL DNA, 1 µL of each primer (100 ng/µL), 12.5 µL of Takara Ex Taq DNA polymerase (Takara Biomedical Technology, Beijing, China), and 10 µL nuclease-free water. PCR conditions were as follows: initial denaturation at 95 °C for 2 min, 35 cycles of denaturation at 95 °C for 30 s, annealing at 49 °C for 30 s, extension at 72 °C for 30 s, with a final extension at 72 °C for 5 min. All PCR products were sequenced by Sangon Biotech Company (Shanghai, China).

The six *D. simplicella* sequences, evaluated using Chromas V2.6.5, were compared with homologous sequences retrieved from the NCBI GenBank database; all sequences were submitted to GenBank, and their accession numbers were obtained (www.ncbi.nlm.nih.gov/GenBank accessed on 22 May 2025). Additionally, 32 *Dioryctria* COI sequences were downloaded from GenBank, and *Pyralis lienigialis* and *Orybina regalis* were used as outgroups. GenBank accession numbers for all sequences analyzed in this study are listed in Table 1. The sequence genetic distances were estimated by the Kimura 2 parameter model (K2P) implemented on MEGA v7.0. To determine the number of haplotypes using DnaSP v6.12, phylogenetic analyses were performed using PhyloSuite v1.2.3. Sequences were aligned using MAFFT v7.526, resulting in a final aligned dataset that included 40 COI sequences of 682 bp. The optimal nucleotide substitution model was determined using ModelFinder. The concatenated sequence matrix was subjected to phylogenetic analysis using both maximum likelihood (ML) and Bayesian inference (BI) methods. The ML analysis was conducted under the TIM + F + G4 nucleotide model with 1000 bootstrap replicates. The BI analysis was performed using Markov chain Monte Carlo (MCMC) sampling algorithm under the GTR + F + G4 nucleotide model and was run for 1,000,000 generations, sampling every 1000 generations, with four chains. The first 25% of trees were discarded as burn-in, and the remaining trees were used to calculate posterior probabilities (PP). The resulting phylogenetic trees were visualized and edited using FigTree v1.4.2.

## 3. Results

### 3.1. Subsection Species Descriptions

*Dioryctria simplicella* Heinemann

*Dioryctria simplicella* Heinemann, 1865: 148–149

Type locality: Germany, Frankfurt am Main

=*Dior. [yctria] abietella* var. *mutatella* Fuchs, 1903: 233

Type locality: Germany—Brandenburg

**Material examined.**

11 males, 16 females, three larvae, Qiqihar, Heilongjiang Province, China, 124.46° N, 48.04° E, 20.V-30VIIII.2024, leg. Niya Jia, laboratory, in *P. sylvestris* var. *mongholica* Litv. cones.

**Diagnosis.** The genital morphology provides reliable diagnostic characters for distinguishing *D. simplicella*. In male genitalia, valva relatively narrow, costa of valva extending distally into a beak-shaped apex bearing a minute spine.
**Description.**

**Figure 1:**
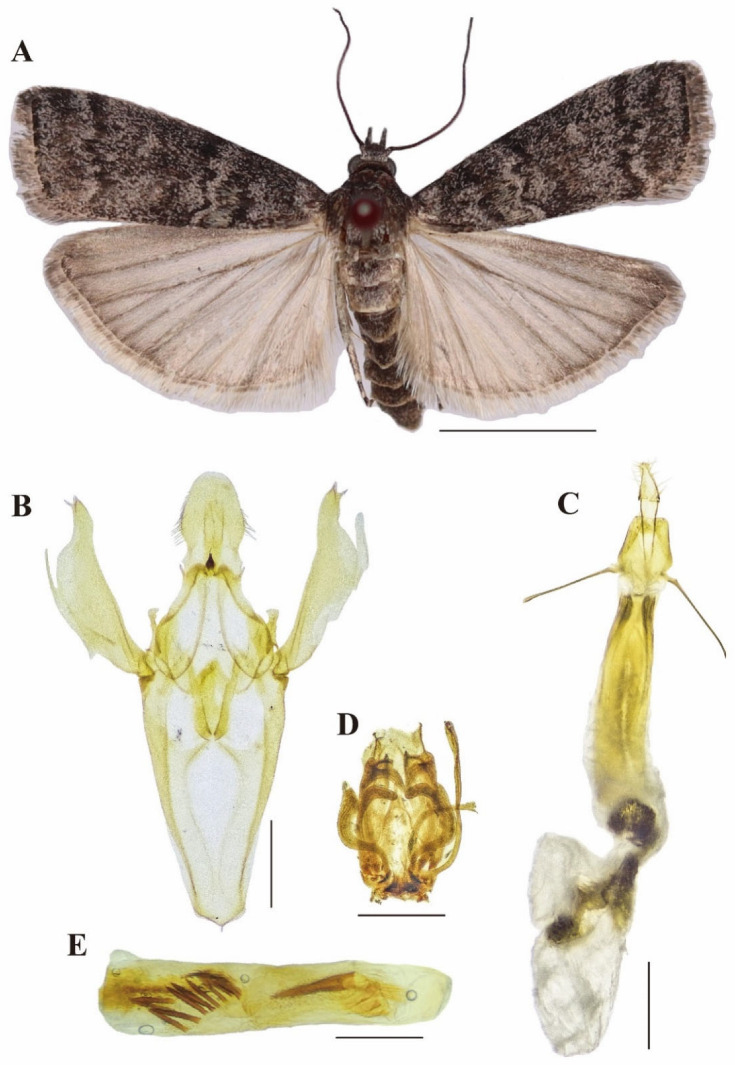
Adult and genitalia of *D. simplicella*. (**A**) adult; (**B**) male genitalia; (**C**) female genitalia; (**D**) male culcita; (**E**) male aedeagus. Scale bars: 5 mm (**A**); 1 mm (**B**–**E**).

Head (Figure 1A). Head black covered with greyish white scales. Antennae grayish brown, filiform, scape black dorsally, flagellum with several basal segments black, the remainders densely short dark brownish cilia. Ocellus glossy yellow. Labial palpus brown, mixed with grayish white; labial palpus 3rd segment ⅓ of length of 2nd segment.

Thorax (Figure 1A). Wingspan 22–30 mm. Thorax blackish mixed with brown white; patagium grayish brown. Forewing grayish brown, suffused with dark brown and grayish white, whitish brown at base and on costa; antemedial line brownish white, broad, sinuate slightly, nearly obsolete near the costa; discal stigma large, brownish white, elliptical; postmedial line brownish white, with two inward angles and one outward angle; terminal line blackish brown, diffused with brownish white along its inside; cilia grayish brown. Hindwing grayish brown; cilia brownish white.

Abdomen (Figure 1A). Abdomen dark brown, covered with grayish brown scales.

Male genitalia (Figure 1B,D,E). Uncus overall appearance egg-shaped; apex rounded; broad at base. Gnathos small, conical, about 1/4 length of uncus. Valva relatively narrow; costa of valva extending distally into a beak-shaped apex bearing a minute spine. Sacculus thin, about 1/3 length of valva. Clasper somewhat triangular. Juxta U-shaped, medially bearing a scutiform sclerite, lateral arms fingerlike, curved inward. Vincullum V-shaped. Aedeagus elongate-cylindrical, internally armed with short spines.

Female genitalia (Figure 1C). Anal papillae short and small, sparsely with setae. Anterior apophyses equal in length to the posterior apophyses, but slightly thicker and with a slightly swollen base. Ductus bursae long and straight, sclerotized, length greater than 4× width, with sclerotized longitudinal stripe in the central third. Corpus bursae membranous, oval. One spine clusters at junction of ductus bursae and corpus bursae, and two groups of spines in upper half of corpus bursae. Ductus seminalis from the 1/3 of ductus bursae.

**Figure 2:**
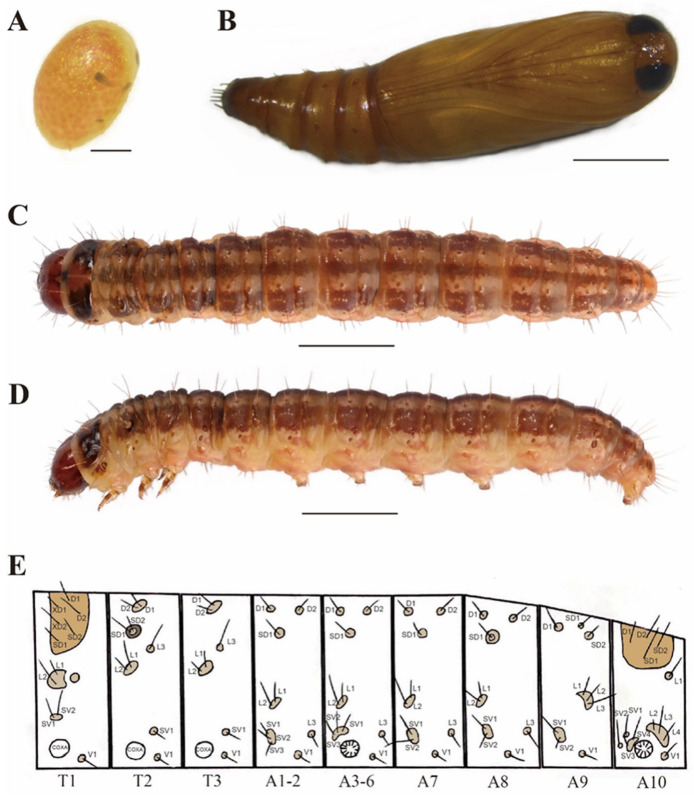
Egg, pupa and larva of *D. simplicella*. (**A**) egg; (**B**) pupa; (**C**,**D**) larva; (**E**) chaetotaxy. Scale bars: 0.1 mm (**A**); 3 mm (**B**–**D**).

Larva (Figure 2C–E). Late instar larvae approximately 16–25 mm long. Head reddish brown. Pronotum dark brown, featuring a median reddish-brown maculation. Body color pale reddish-brown, dorsal surface of abdomen bearing three parallel dark brown longitudinal stripes extending from the cephalic to the caudal end, cuticle smooth. Chaetotaxy as shown in Figure 2E.

Thorax Segment (T1–T3). T1: D1 closer to XD1 than to D2; XD2, SD1, and SD2 are approximately equilateral triangles; L group bisetose; SV1 and SV2 are present above the leg; T2 and T3: D1 and D2 on a pinaculum; L group trisetose, L1 and L2 are clustered on a single pinaculum; SV group unisetose; SD1 and SD2 are present on T2, a dark ring around seta SD1 and SD2.

Abdomen Segment (A1–A10). On A1–A8, D group bisetose; SD group unisetose; L group trisetose, L1 and L2 are clustered on a single pinaculum; SV group trisetose on A1–A6; SV group bisetose on A7 and A8; a dark ring around seta SD1 and the center is pale on A8. On A9, D group unisetose; SD group bisetose; L group trisetose and on a single pinaculum; SV group bisetose. On A10, D1 and D2, SD2 and SD1, all coalesced on a shared pinaculum; L and SV group quadrisetose; L2–L4 are clustered on one pinaculum; SV3 and SV4 share a pinaculum.

Egg (Figure 2A). The egg is elliptical in shape and approximately 0.8 mm in length, pale yellow when freshly laid, with scattered red spots gradually appearing on the surface as embryonic development progresses.

Pupa (Figure 2B). The obtect pupa is spindle-shaped, with a body length of 8–12 mm, yellowish-brown in color, turning dark brown prior to eclosion, with six caudal spines at the posterior end of the pupa.

Distribution. China (Heilongjiang, new record), Germany, Bulgarian, Belarus, England, Austria, Belgium, Denmark, Czechia Republic, Estonia, Finland, France, Hungary, Latvia, Lithuania, Luxembourg, Netherlands, Norway, Romania, Slovakia, Sweden, and Switzerland.

Ecology. *D. simplicella* has one generation per year in Heilongjiang, China. Adults emerge from the end of May to the middle of September.

**Figure 3:**
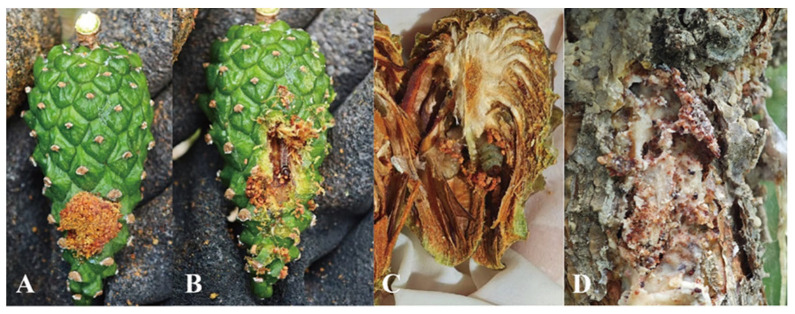
*P. sylvestris* var. *mongholica* Litv. cone and trunk damaged by *D. simplicella* larvae. (**A**) external view of an infested cone; (**B**) larva inside the cone; (**C**) cone damaged by a mature larva in October; (**D**) infested trunk.

Host (Figure 3). *P. sylvestris* var. *mongholica* Litv. (New to China), *Pinus sylvestris* [24,31], and *Picea glauca* [24].

### 3.2. Molecular and Phylogenetic Analyses

**Figure 4:**
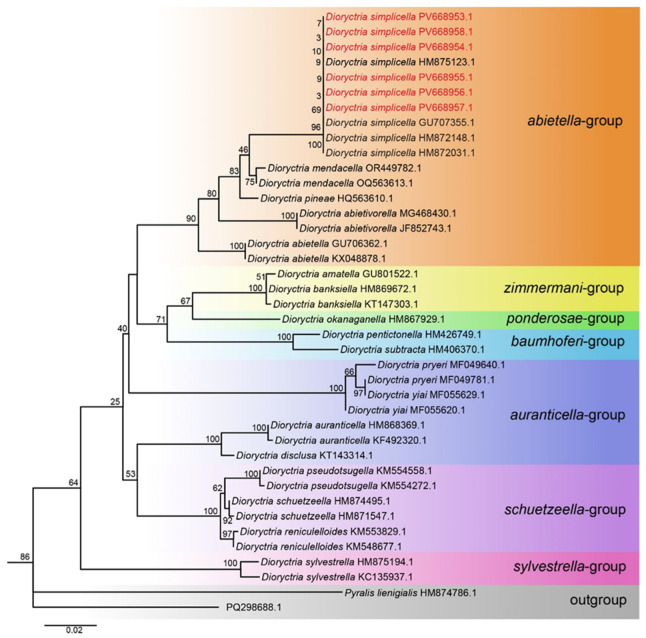
Maximum likelihood (ML) phylogenetic trees constructed based on mtDNA COI.

We successfully obtained and sequenced 682 bp COI fragments from six *D. simplicella* specimens (two larval, two male adults, and two female adults). The final dataset comprised 40 COI sequences representing 20 species across three genera within the Pyralidae family (Table 1). No intraspecific variation (genetic distance = 0) was observed among the 10 *D. simplicella* sequences analyzed in this study. The smallest interspecific distance involving *D. simplicella* was found with *D. mendacella* (0.021). Genetic distances between *D. simplicella* and other *Dioryctria* species ranged from 0.021 to 0.109, with a mean distance of 0.068. The detailed pairwise genetic distance matrix is presented in the Supplementary Materials: Table S1. Haplotype analysis conducted in DnaSP v6.12 revealed a single haplotype among the 10 *D. simplicella* sequences. Both maximum likelihood (ML) (Figure 4) and Bayesian inference (BI) (Supplementary Materials: Figure S1) analyses of the COI dataset yielded congruent topologies, strongly supporting the monophyly of *D. simplicella*. The phylogenetic trees placed *D. simplicella* within the *abietella*-group. Consistent with the 2% threshold proposed by Hebert et al. [26] for species delimitation using COI barcode sequences, the observed genetic distances support the distinct status of *D. simplicella*. Overall, the results of the phylogenetic analysis corroborate the morphological identification of *D. simplicella*.

## 4. Discussion

This study reports the discovery of *D. simplicella* in China. We found *D. simplicella* larvae in the trunks and green cones of *P. sylvestris* var. *mongholica* Litv., captured adults through light trapping in the field, and obtained eggs and pupae under laboratory conditions. Both adult and larval specimens were identified to the species level through morphological and molecular biological methods, confirming them as *D. simplicella*.

Accurate identification of *D. simplicella* requires comprehensive consideration of geographical distribution and ecological information. According to reports, adult *D. simplicella* exhibit two morphs: the grey and the black. The black morph can be rapidly distinguished by its black forewing coloration [23,32], while the grey morph more closely resembles the typical wing pattern of most *Dioryctria* species [10,24]. The *D. simplicella* specimens discovered in China belong to the grey morph. Recent reports also confirm the grey morph in Belarus [33]. *D. simplicella* is widely distributed across Europe, where the occurrence frequency of the grey is significantly higher than that of the black. Initially, Hassler and Speidel [34] considered the black *D. simplicella* endemic to Germany; however, subsequent research revealed its distribution extends far beyond southwestern Germany and is widespread across Europe. In British populations, the black can comprise up to 25% of individuals [35]. Molecular evidence supports that the grey and black morphs of *D. simplicella* represent a single species. These morphs do not form distinct clusters corresponding to their phenotypes but instead jointly form a terminal polytomy, characteristic of a typical monophyletic group [16]. In the present study, *D. simplicella* specimens from China clustered closely with European *D. simplicella* from NCBI on the phylogenetic tree, supporting the conspecificity of Eurasian populations as *D. simplicella*. The *D. simplicella* larvae primarily feed on green cones, shoots, and buds of *P. sylvestris* and *P. glauca* [24,31]. The population of *D. simplicella* in China damaged not only these plant parts but also the main trunk, inducing substantial resin accumulation on trunks (Figure 3D).

The present study extends the geographic distribution of *D. simplicella* to East Asia and provides new data supporting taxonomic and phylogenetic research on the subfamily Phycitinae. This discovery enriches the diversity records of Chinese Lepidoptera. Our surveys confirm the extensive distribution of *D. simplicella* across *P. sylvestris* var. *mongolica* stands in Northeast China. This species poses a latent threat to pine health in Northeast China. Our study establishes fundamental data essential for the monitoring, management, and further taxonomic studies of *D. simplicella* in China.

## Figures and Tables

**Table 1:** Samples of COI genes used in this study.

Species Group	Species	GenBank NO.
*abietella*-group	*Dioryctria simplicella*	PV668953.1
	*Dioryctria simplicella*	PV668954.1
	*Dioryctria simplicella*	PV668955.1
	*Dioryctria simplicella*	PV668956.1
	*Dioryctria simplicella*	PV668957.1
	*Dioryctria simplicella*	PV668958.1
	*Dioryctria simplicella*	HM875123.1
	*Dioryctria simplicella*	HM872148.1
	*Dioryctria simplicella*	HM872031.1
	*Dioryctria simplicella*	GU707355.1
	*Dioryctria abietella*	GU706362.1
	*Dioryctria abietella*	KX048878.1
	*Dioryctria abietivorella*	MG468430.1
	*Dioryctria abietivorella*	JF852743.1
	*Dioryctria mendacella*	OR449782.1
	*Dioryctria mendacella*	OQ563613.1
	*Dioryctria pineae*	HQ563610.1
*zimmermani*-group	*Dioryctria amatella*	GU801522.1
	*Dioryctria banksiella*	HM869672.1
	*Dioryctria banksiella*	KT147303.1
*auranticella*-group	*Dioryctria auranticella*	HM868369.1
	*Dioryctria auranticella*	KF492320.1
	*Dioryctria disclusa*	KT143314.1
	*Dioryctria pryeri*	MF052103.1
	*Dioryctria pryeri*	MF049781.1
	*Dioryctria yiai*	MF055629.1
	*Dioryctria yiai*	MF055620.1
*ponderosae*-group	*Dioryctria okanaganella*	HM867929.1
*baumhoferi*-group	*Dioryctria pentictonella*	HM426749.1
	*Dioryctria subtracta*	HM406370.1
*schuetzeella*-group	*Dioryctria pseudotsugella*	KM554558.1
	*Dioryctria pseudotsugella*	KM554272.1
	*Dioryctria reniculelloides*	KM553829.1
	*Dioryctria reniculelloides*	KM548677.1
	*Dioryctria schuetzeella*	HM874495.1
	*Dioryctria schuetzeella*	HM871547.1
	*Dioryctria sylvestrella*	HM875194.1
	*Dioryctria sylvestrella*	KC135937.1
outgroup	*Pyralis lienigialis*	HM874786.1
outgroup	*Orybina regalis*	PQ298688.1

## Data Availability

The original contributions presented in this study are included in the article/Supplementary Materials. Further inquiries can be directed to the corresponding authors.

## Supplementary Materials

The following supporting information can be downloaded at https://www.mdpi.com/article/10.3390/insects16070664/s1, Figure S1: Bayesian inference (BI) phylogenetic trees constructed based on mtDNA COI; Table S1: Intraspecific and interspecific genetic distances of *Dioryctria* species in this study.
