# Supplementary material for: First Record of Dioryctria simplicella (Lepidoptera: Pyralidae) in China: Morphology, Molecular Identification, and Phylogenetic Position
Source: Insects. 2025 Jun 26;16(7):664. doi: 10.3390/insects16070664 (PMC12295827; doi:10.3390/insects16070664)
Supplement: Supplementary file 1 [file Figure S1.pdf › Figure S1.pdf]

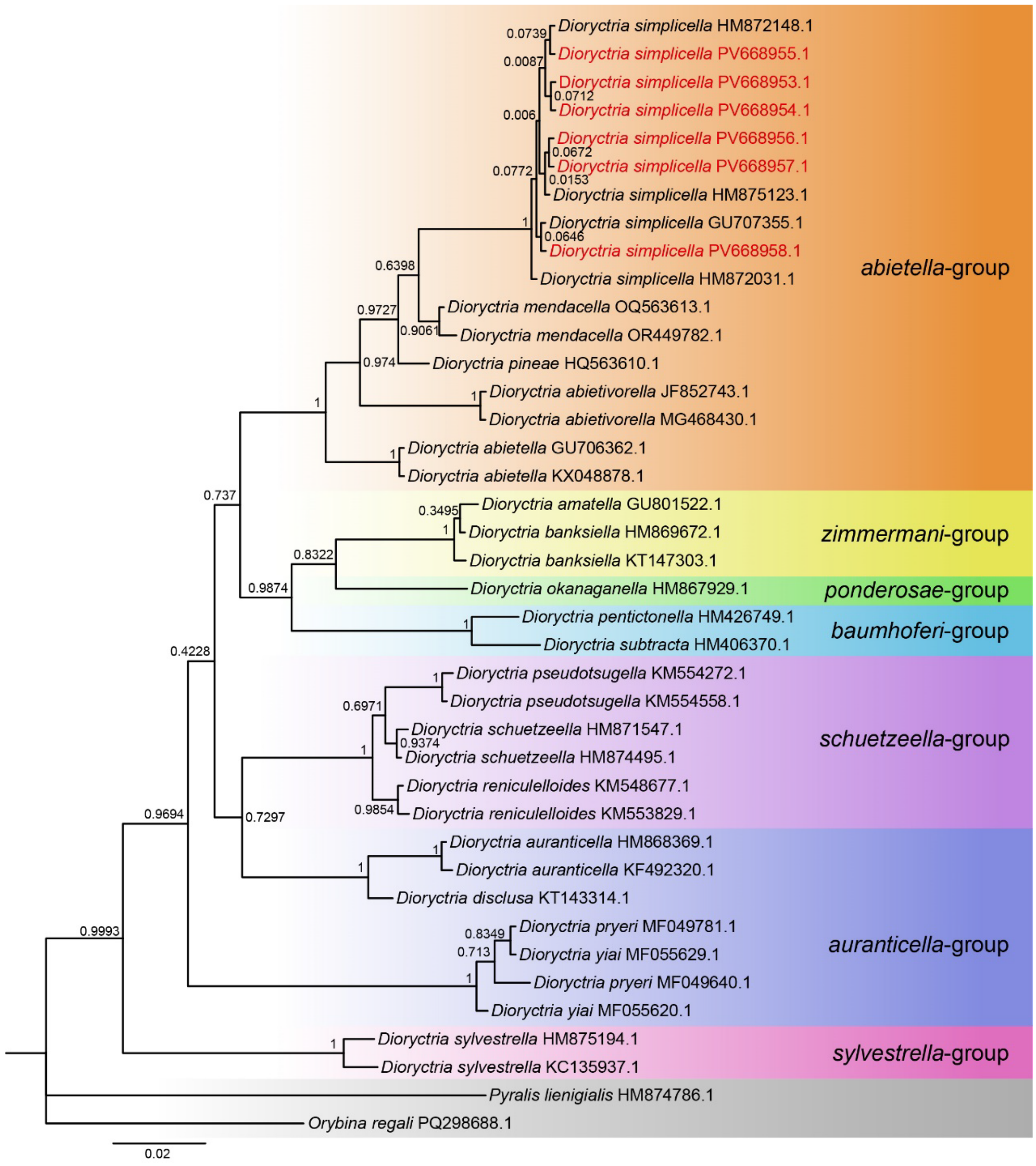


**Figure S1.** Bayesian inference (BI) phylogenetic trees constructed based on mtDNA COI.
